# Facing up to disability

**Published:** 2013

**Authors:** Tom Shakespeare

**Affiliations:** Senior Lecturer in Medical Sociology: University of East Anglia, Norwich, UK.

**Figure:**
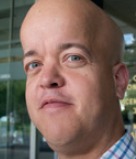
Tom Shakespeare

Ways of thinking about and responding to disability have radically changed in recent decades. Traditionally, disability was regarded in terms of sin, karma, or divine punishment. More recently, disability was made a medical issue and defined in terms of shortcomings of body or mind, which had to be prevented or cured at all costs. In the late 20th century, people with disabilities worldwide became more organised and created national and international disabled people's organisations. They successfully demanded that disability be seen as a matter of equal opportunities and human rights, a shift which has now been described in the United Nations Convention on the Rights of Persons with Disabilities. This is a global treaty which has so far been signed by 155 states and passed into law by 127.

Disabled activists and academics make a distinction between **impairment** – in the individual's functioning – and **disability**, understood as the relationship between a person with impairment and their society. By failing to consider the needs and wants of people with impairments, and failing to make the world more accessible for them, society is in fact responsible for disabling people who have impairments. This is known as the social model of disability.

Disability is shaped by **physical barriers** (e.g. medicine labels which are too small for people with visual impairment to read, or stairs to the hospital entrance which prevent people using wheelchairs from going in) and **social barriers**, including negative attitudes and cultural messages, and discrimination in employment.

**Figure:**
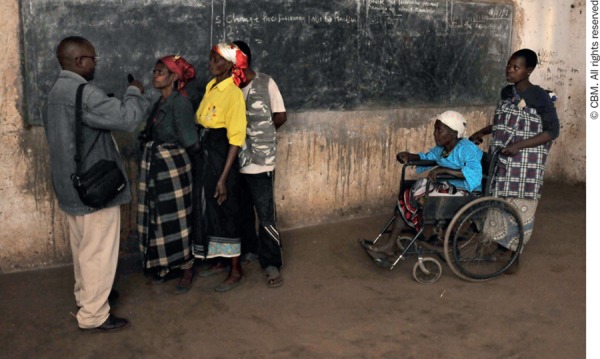
Community-based rehabilitation workers can take eye care into the community, thereby increasing access for people with different impairments. MALAWI

**ABOUT THIS ISSUE**

**Figure:**
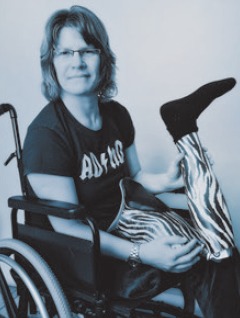
Diane Mulligan

Deputy Director, Advocacy and Alliances for Inclusive Development: CBM International. diane.mulligan@cbm.org

I am delighted to have been asked to be the consulting editor for this special issue on disability and diversity. It covers a range of interesting articles that are relevant for anybody working in community eye health specifically, as well as in the health sector generally. As an amputee myself, I often find myself making jokes to put people at ease around me. Why? Simply because most people are not confident about how to approach or interact with people with disabilities, and humour breaks down barriers. This journal is jam-packed full of articles that will give you information to boost your confidence. In particular, the section on practical tips for eye care workers on how to engage with people with different impairments, and the poster on guiding someone who is blind, are simple and straightforward.

The editorial, written by Professor Tom Shakespeare, until recently working on disability with the World Health Organization, gives a great overview. ‘What does it mean to have an impairment?’ is a wonderful interview with Gertrude Fefoame, a blind Ghanaian disability advocate and mother of three. Read about her brilliant insights and powerful solutions to barriers she has faced.

The moving story of disability from a child's perspective is captured by Maria Zuurmond's article. It demonstrates how we need to work in the most inclusive way possible for the next generation.

The articles that follow are full of practical ideas about how to make eye care inclusive and accessible. You'll find some key recommendations, an overview of what inclusion, participation and accessibility actually mean; and a case study from Cambodia.

And the artices on community-based rehabilitation and disabled persons' organisations emphasise the importance of referral to services both inside and outside of the health system.

Read on, and enjoy!

## Stereotypes

Stereotypes influence the ways that people without disabilities react to people with disabilities. For example, people with disabilities are sometimes considered to be childlike and innocent, and are spoken down to. People with disabilities are thought of as dependent and incompetent. Yet, in reality, people with disabilities are like everyone else, with strengths and weaknesses. Throughout recorded history, presidents, military leaders, writers, artists, musicians, sportspeople, and scientists have had impairments, showing that this is no bar to high office or great achievement in life (see www.disabledlives.blogspot.com).

People with disabilities are more likely to be female, older, and/or poorer. When people with disabilities are also of minority ethnic status or gay, their difficulties are even more complex, leading to greater disadvantage. People with certain impairments are also more excluded than others – for example, people with intellectual impairments and people with mental health conditions are particularly disadvantaged in employment.

**Glossary: disability**

- **Accessibility**. The degree to which information, a service, or a device/product is available to as many people as possible, including people with different impairments.
- **Barriers**. Those things which prevent a person with an impairment from being able to get to, or use, information, services, or devices/products.
- **Disability**. How an impairment affects someone's life; this is determined by the extent to which society is willing to accommodate people with different needs.
- **Inclusion**. The practice of ensuring that people feel they belong and are able to participate in community life, which includes accommodating any person with an impairment.
- **Intellectual disability**. A reduced intellectual ability and difficulty with everyday tasks; the term ‘mental disability’ is similar but can include mental disorders such as depression or schizophrenia. Other terms used for intellectual disability include ‘learning disability’ and ‘mentally handicapped’.
- **Impairment**. Difficulty in physical, mental or sensory functioning.
- **Mobility impairment**. Difficulty with walking or moving around. People with mobility impairments may be wheelchair users or use crutches, or may need extra time or support from another person to move around.
- **Sensory impairment**. Visual and/or hearing impairments.

How then to improve the quality of life of people with disabilities? *The WHO/World Bank World Report on Disability*, launched in 2011 as an evidence-based summary of the global situation of people with disabilities, provides information on problems and solutions. Rather than reducing disability to simply medical problems and solutions, the social approach used in the report highlights how social arrangements, policies, and practices can either disable or enable people with impairments. For example, failure to provide rehabilitation and assistive devices means that people remain dependent and cannot improve their functioning. If children with disabilities are not able to attend school, they may be denied the chance of future employment. Inaccessible transport means that people with mobility impairments cannot access health care, education, or employment.

The way forward is clear. As mandated by the United Nations Convention on the Rights of Persons with Disabilities, governments should promote access to health, education, livelihood and other opportunities. People with disabilities should live in the community, rather than institutions. In general, the best strategy is removing barriers by making physical changes and by working to improve the attitudes and skills of staff. This will ensure that health care and other services are available and accessible to all. Sometimes, it is necessary to provide additional services specifically for people with disabilities, to ensure they can benefit from the specific services they require, for example rehabilitation or support.

Eye care practitioners should examine their own practices to ensure that they treat people with disabilities in the same way as they treat other people. After all, people with disabilities have the same needs, are vulnerable to all the same risks, and require the same access to mainstream services as other people. In busy clinics, eye care practitioners may think only about the person's eye health and forget about the person's general health and their other needs, for example education, rehabilitation, or support with getting a job.

People with disabilities are entitled to respect and dignity, and should be treated fairly. Service providers – such as eye clinics – must be flexible and willing to adapt environments and practices to accommodate different ways of working, communicating, or moving around.

Prevention of disabling health conditions of course remains a priority for global development and public health. But for the millions of people worldwide who live with impairment, their best hope of flourishing lies in a society which is committed to the principle of inclusion and willing to remove any barriers in their way.
